# Chromium "(VI)" phytoremediation using *Azolla pinnata*: effects on *Vicia faba* growth, physiology, cytogenetics, and gene expression profiling

**DOI:** 10.1186/s12870-025-06115-7

**Published:** 2025-02-07

**Authors:** Elham R. S. Soliman, Kareem Moustafa, Mohamed Khamis, Zeinab A. Shedeed

**Affiliations:** 1 Botany and Microbiology Department, Faculty of Science, Helwan University, Helwan, 11795 Egypt; 2 Molecular Biotechnology Program, Chemistry Department, Faculty of Science, Helwan University, Helwan, 11795 Egypt

**Keywords:** Chromosomal aberrations, DNA degradation assay, Genotoxicity, Germination, Mitotic index, Oxidative stress, Potassium dichromate, QRT-PCR

## Abstract

**Background:**

One of the primary challenges that the expanding population faces is water scarcity. Thus, a global imperative has been established to safeguard extant water resources and optimize their utility through sustainable practices and efficient management. In the present investigation, *Azolla pinnata*, a pteridophyte (fern), was employed to phytoremediate Cr (VI) from chromium-polluted water. The potential of this treated water for agricultural purposes was verified through the use of *Vicia faba* plants.

**Results:**

In vitro, *A. pinnata* effectively remediates Cr (VI) from an array of liquid concentrations (0.05 to 90 ppm) in a ratio of 25:1 {volume (mL): fresh weight of *Azolla* (g)} after 2 days incubation period at room temperature. At low concentrations (0.1 ppm), the phytoremediation capacity peaked at 70%, falling to 19.53% at a high concentration (90 ppm). Upon continuous irrigation with Cr-polluted water (0.05 to 50 ppm), the in vivo pot experiment on *Vicia faba* plants revealed high Cr accumulation in the roots reached 52.5 mg Kg^-1^ dry weight (Dwt) at the 50 ppm Cr treatment. Nevertheless, a reduced Cr content of 19.5 mg Kg^-1^ Dwt was observed when the plants were irrigated with 50 ppm Cr-polluted water that had been treated with *Azolla*. At 50 ppm of Cr, *Azolla's* treatment significantly increased shoot length, fresh weight, and Chl a content to 25.25 cm, 3.4 g, and 6.5 mg g^-1^ Dwt, respectively, up from 10.25, 1.8, and 4.7 in untreated plants. The chromosomal aberrations were significantly induced in the dividing cells of all Cr treatments, with the highest value of 4.8% at 50 ppm. This value was reduced to 2.88% at the same concentration when treated with *Azolla*. At a concentration of 10 ppm Cr, the mitotic index was significantly improved to 6.99% when combined with *Azolla*, as opposed to 3.63% when the same concentration was used without *Azolla*. The DNA degradation assay showed partial DNA degradation at 50 ppm Cr, which the *Azolla* treatment eliminated. Furthermore, the gene expression levels of both the PM H^+^-ATPase and the calcium-dependent protein kinase CDPK5 were upregulated in response to Cr, despite the fact that the expression level was altered in a dose- and concentration-dependent manner by *Azolla* treatment.

**Conclusion:**

*Azolla* exhibits substantial potential for reducing the detrimental effects of chromium stress including oxidative stress on plants. It modulates stress-related gene expression, protects DNA integrity, enhances cell mitosis, and reduces chromosomal damage. These results indicate that *Azolla* has the potential to be a valuable asset in phytoremediation strategies for chromium-contaminated environments, and that it may enhance plant survival and growth under Cr stress conditions.

**Key message:**

*Azolla pinnata* can be effectively utilized as an environmentally-friendly method to remediate chromium-contaminated water for agricultural usage.

## Highlights

• *A. pinnata* remediates Cr(VI) from liquid concentrations with 70% phytoremediation capacity at low concentrations.

• *A. pinnata* treatment greatly decreased Cr buildup and improved *Vicia faba* growth under Cr stress.

• In *Vicia faba*, *A. pinnata* increased mitotic index, reduced Cr-induced chromosomal aberrations, and modulated the expression of genes linked to stress.

## Introduction

Chromium (Cr) is an element which naturally occurs in water and soil. Cr (VI) pollution is closely related to various Cr (VI) industrial applications such as electroplating, dyeing of textiles, leather processing, steel production and tanning industry, causing its release in industrial effluents [1]. Because of its high solubility, Cr (VI) is considered a dangerous ion that can pass through the food chain [1]. List of hazardous substances, from Agency of Toxic Substances and Disease Registry (ATSDR) (2019), ranks chromium (Cr) as one of the most dangerous chemicals (the 17th) [2].

For plants, Cr is considered to be toxic for growth, hindering their vital metabolic progressions [3]. Exposure to Cr has deleterious influences on seed germination, as well as morphological and biochemical characteristics [3, 4]. As a redox active metal, Cr induces the production of reactive oxygen species (ROS), such as H_2_O_2_ and O_2_-, as well as MDA in plants, leading to damage in DNA, RNA, proteins, and pigments [5]. This results in oxidative stress, which disturbs the cellular equilibrium between oxidants and antioxidants. In order to maintain this equilibrium within the cell, enzymatic and non-enzymatic antioxidants scavenge the reactive oxygen species produced [6]. Numerous plant species, such as *Vigna radiata* [7], *Brassica juncea* [8], *Cicer arietinum* [9], *Sorghum bicolor* [10], and *Zea mays* [11] have been subjected to investigations into the cytotoxic effects of Cr. Chromium oxide induces modifications in the structure of DNA, potentially including strand cross-linking and condensation. The primary catalyst for these reactions is the intercalation of Cr elements into the pairs of DNA bases. Consequently, chromium has the potential to disrupt DNA replication, transcription, and function, all of which are critical for the transmission of genetic information [12]. Chromium accumulation in plants inevitably causes disturbances to their homeostasis, leads to an increase in the frequency of chromosome distortion in root apex cells, and diminishes the volume and number of root cells [13].

There are no specific genes identified as direct targets of chromium stress. However, with the advancement of molecular biology, some genes connected to chromium resistance have been identified. The gene expression patterns of molecular transporters, ion binding protein kinases, and metal transition-related genes (BnaA04g26560D, BnaA02g28130D, and BnaA02g01980D) may be altered in plants subjected to chromium stress [14]. Significant upregulation of the metallothionein (MT) gene was observed in both the stem and leaf in response to chromium stress. This MT protein is capable of metal binding and metal ion detoxification [15]. The inheritance of heavy metal uptake and translocation in plants is facilitated by the transgenerational memory of the P1B subfamily of heavy metal-transporting P-type ATPases (HMAs), according to a discovery of Cong et al. [16].

*Azolla*, a macrophyte fern that floats freely, is a ubiquitous species that can endure both freshwater and effluent environments. The biomass of this organism has the capacity to double every 2–5 days, and it is especially effective at concentrating hazardous heavy metals [17]. In the pursuit of sustainable methods to conserve water as a renewable resource, it was discovered that *Azolla* could serve as a viable and practicable instrument for preserving the quality of irrigation water through the accumulation of contaminants. Sharma et al. [18] demonstrated in a recent study that *Azolla filiculoides* was capable of bioaccumulating a diverse array of heavy metals, including Fe, Si, Cr, Ni, Cu, Pb, Zn, Al, Mn, Cd, and B, to varying degrees depending on the heavy metal type and concentration. Furthermore, Naghipour et al. [19] demonstrated that the efficacy of *Azolla filiculoides* in removing Ni (II), Cd (II), and Pb (II) was enhanced when the contact time was extended to 10 days, but declined when the metal concentration was increased from 5 to 25 mg/L. *Azolla filiculoides* phytoremediated six heavy metals (Cr, Co, Ni, Zn, Cd, and Pb) from the effluent of a soft drink factory, with the highest removal rate of 90.04 percent for Cr [20]. While Sharma et al. [18] and Naghipour et al. [19] demonstrated *Azolla filiculoides'* ability to bioaccumulate various heavy metals and studied the impact of heavy metal accumulation on *Azolla*'s growth characteristics, little is known about the direct effect on plants that were irrigated with *Azolla*-remediated water for sustainable agriculture purposes. Therefore, a complete investigation incorporating the morphological, physiological, genetical, and molecular responses of the plants that were irrigated with *Azolla*'s treated heavy metal-contaminated water would be beneficial in appraising a new phytoremediation strategy for heavy metal-contaminated habitats.

Consequently, the objective of the present investigation is to assess the efficacy of *A. Pinnata* as a phytoremediant for chromium removal from chromium contaminated water. Utilizing *Vicia faba* plants, the feasibility of recycling this treated water for agricultural purposes was confirmed. The concentrations of Cr and K in the shoots and roots of plants irrigated with *Azolla*-treated Cr-water as well as those not treated with *Azolla* were determined. An evaluation was conducted on the growth parameters, certain physiological characteristics, cytotoxicity, genotoxicity and gene expression analysis of PM-type H+-ATPases, and CDPK5 genes.

## Materials and methods

### Phytoremediation of chromium (VI)-contaminated water with *A. pinnata*

Different concentrations of Cr were prepared from anhydrous K_2_Cr_2_O_7_ ranged from (0.05 to 90 ppm Cr). Fifty milliliters of each concentration were prepared using deionized water (pH: 7) and supplemented with 2 g of fresh *A. pinnata* that kindly purchased from Agriculture research center (ARC), Giza, Egypt. The solutions were left at room temperature (25 °C) under normal day/night regime in colorless bottles to allow sunray penetration. After two days the solutions were filtrated to get rid of *A. pinnata*. The Cr concentration was measured in the clear filtrates using Agilent 4210 MP-AES. The phytoremediation efficiency (*E*) was calculated as follow: $E=\frac{\text{C}1-\text{C}2}{\text{C}1}\times 100,$ Where C_1_, is the initial concentration of Cr, C_2_, the final concentration of Cr after phytoremediation.

### Instrumentation

The solutions for Cr were analyzed using Agilent 4210 MP-AES (Microwave Plasma-Atomic Emission Spectrometry, Agilent Inc.) at Ecology laboratory, Faculty of Science, Helwan University. This instrument was equipped with a quartz torch, a glass concentric nebulizer and a cyclonic spray chamber. The viewing position and nebulizer flow were optimized every time using the MP-AES. Calibration aqueous solution was used for internal instrument calibration. Five aqueous standards were prepared from commercial elemental Merck IV solution in ppm of measured element calibration methodology. The recovery rate of the instrument is from 95 to 110% according to the element based on manufacturer. The analysis was performed in triple replicates.

### Pot experiment on *V.faba* plants and evaluation of vegetative growth attributes

The experiment was implemented in plastic pots (10 cm diameter). The pots contained 2 Kg clay for cultivation. The experiment was performed in triplicates at Faculty of Science, Helwan University during winter 2023 under natural conditions. Five seeds of beans (*Vicia faba* L.) were cultivated in each pot. The experiment was divided into two groups: the 1^st^ group was irrigated with different concentration of Cr (VI) (0.05, 10, 30, 50 ppm) using anhydrous K_2_Cr_2_O_7,_ the 2^nd^ group was irrigated with the same different concentration of Cr (0.05, 10, 30, 50 ppm) post *A. pinnata* treatment for 2 days. The seeds were cultivated in soil and irrigated regularly at three days interval. For comparison a control group was irrigated with tap water. The plants were harvested after 38 days of cultivation in soil to measure the vegetative and growth parameters. Each plant was divided into shoot and root, their fresh weights (g) and lengths (cm) were measured (4 replicates). Then, they were oven dried at 60 °C for 2 days to measure their dry weights (g). Leaves number per each plant were also recorded for each treatment.

### Determination of Cr and K concentrations in *V. faba* plants

For Cr and K analysis in plant tissue, the protocol recommended by Oliva & Rautio [21] and Ukpebor et al. [22] was used. The washed and dry shoot and root samples (0.5 g) were ashed at 550 ºC in a muffle furnace for 3 h and then digested with 10 mL nitric acid (2.8%) overnight. The sample volume was brought to 50 mL using deionized water. The solutions were analyzed for Cr and K using Agilent 4210 MP-AES (Microwave Plasma-Atomic Emission Spectrometer, Agilent Inc.) as described above. The analysis was performed in triple replicates.

The translocation factor (TF) was calculated according to Takarina et al. [23].

$$\mathrm{TF}=\frac{\mathrm{Cr}\;\mathrm{concentration}\;\mathrm{in}\;\mathrm{shoot}}{\mathrm{Cr}\;\mathrm{concentration}\;\mathrm{in}\;\mathrm{root}}$$

### Evaluation of the physiological attributes of Cr-stressed *V. faba* plants

#### Photosynthetic pigment content

Weighted 0.5 g fresh *V. faba* leaves were homogenized in diluted acetone (85%) [24]. The homogenate was centrifuged at 4 °C. Then, the supernatant was completed to 10 mL using acetone and measured at 645 nm, and 664 nm wavelengths against blank. The analysis was performed in triple replicates.

Then the concentration of chlorophyll (a and b) was calculated according to the following formula,

$$\mathrm{Chlorophyll}\;\mathrm a=10.3\;\mathrm E664-0.918\;\mathrm E645,\;\mathrm{Chlorophyll}\;\mathrm b=19.7\;\mathrm E645-3.87\;\mathrm E664$$

#### Antioxidant enzymes extraction

Half gram of fresh *V. faba* leaves was ground with 10 mL of cold phosphate buffer (0.2 M, pH 6.2). This mixture was filtered through cheesecloth, then centrifuged for 10 min at 6000 rpm at 4°C [25]. The preserved filtrate was used for enzyme assay as follows.

##### Catalase bioassay

Catalase (EC 1.11.1.6) was assayed according to Góth [26] by using ammonium molybdate. An 0.2 mL of the plant extract was mixed with 1 mL of 65 mM hydrogen peroxide prepared in 60 mM Na/K phosphate buffer. After incubation for 4 min at 25 ºC, 1 mL of ammonium molybdate was added to stop the reaction. The intensity of the developed yellow complex of molybdate and hydrogen peroxide was measured at 405 nm. The analysis was repeated for three replicates.

##### Peroxidase bioassay

Peroxidase (EC.1.11.1.17) activity was measured by Guaiacol method according to Angelini et al. [27]. The reaction mixture contained 2.2 mL of 0.1 M potassium phosphate buffer (pH 7.0), 0.5 mL of 0.018 mM guaiacol, 0.2 mL of 30% H_2_O_2_. Immediately after adding 0.1 mL of the crude enzyme extract to the reaction mixture, the initial absorbance measured (at zero time). Then, record absorbance measurements every 30 secs for 3 minutes (6 readings) at 436 nm, following the changes in absorbance in specific weight and time.

$$\mathrm{Calculate}\;\mathrm{the}\;\mathrm{change}\;\mathrm{in}\;\mathrm{absorbance}\;(\triangle\mathrm A)\;\mathrm{for}\;\mathrm{each}\;\mathrm{sample}:\;\triangle\mathrm A=\mathrm A\;\mathrm{Final}-\;\mathrm A\;\mathrm{Initial}$$

### The total antioxidant capacity

Prieto et al. [28] method was used to determine the total antioxidant capacity of the plant extract in triplicates samples. 1.0 mL of the reagent solution (0.6 M sulphuric acid, 28 mM sodium phosphate and 4 mM ammonium molybdate) was added to 0.5 mL of the plant extract. The tubes were mixed and incubated at 95°C water bath for 90 mins. After incubation, the tubes were cooled and the absorbance was measured at 695 nm against a blank. Ascorbic acid was used as standard and the total antioxidant capacity is expressed as equivalents of ascorbic acid.

### Cytogenetic evaluation

The roots were collected from each treatment at early morning and thoroughly rinsed in running water to wash out the soil particles then fixed in Carnoy's fixative (3 ethyl alcohol: 1 glacial acetic acid{v/v}) and stored in refrigerator at 4°C for further use. The harvested root tips were stained in Feulgen stain after their hydrolyzation in 1N HCl at 60°C for 10 min [29]. The stained root tips were squashed in 45% acetic acid on a slide glass then the slides were observed and images were captured from the light microscope (OLYMPUS CX31 microscope and photographed with ToupCam) at both 400× and 1000× magnification power. At least, 15 independent root tips were examined for each treatment and >3000 cells were considered for mitotic index count. A total number of 5301, 3264, 3066, 5688 and 5741 cells were scored for control, 10 ppm Cr, 10 ppm Cr + *Azolla*, 50 ppm Cr and 50 ppm Cr + *Azolla* treatments, respectively. In addition to cell count scoring, each observation field was examined to determine the mitotic phase and type of chromosomal abnormality. The chromosomal aberrations were scored as a type as follow: erosion, fragmentation, hyperchiasma, polyploidy at interphase; uncoil, breaks hyperchiasma at prophase; stekinesis, breaks, C-metaphase, stickness, at metaphase; disturbance, breaks, bridge, stekinesis, nonpolar polarization, stickness at both anaphase and telophase. These data were used to calculate the mitotic index (MI) and different mitotic parameters according to the follow:

$$\mathrm{Mitotic}\;\mathrm{index}\;\left(\mathrm{MI}\right)=\frac{\mathrm{Total}\;\mathrm{number}\;\mathrm{of}\;\mathrm{divided}\;\mathrm{cells}\;\mathrm{counted}\;\mathrm{at}\;\mathrm{each}\;\mathrm{treatment}}{\mathrm{Total}\;\mathrm{number}\;\mathrm{of}\;\mathrm{cells}\;\mathrm{at}\;\mathrm{each}\;\mathrm{treatment}}\times100$$

$$\%\;\mathrm{of}\;\mathrm{total}\;\mathrm{chromosomal}\;\mathrm{aberrations}=\frac{\mathrm{Total}\;\mathrm{number}\;\mathrm{of}\;\mathrm{aberrant}\;\mathrm{cells}\;}{\mathrm{Total}\;\mathrm{number}\;\mathrm{of}\;\mathrm{cells}}\times100$$

*"Total number of aberrant cells" refers to the number of cells that exhibit a type of aberration at interphase and any dividing stage (prophase, metaphase, anaphase, and telophase).*

$$\%\;\mathrm{of}\;\mathrm{dividing}\;\mathrm{chromosomal}\;\mathrm{aberrations}\;\left(\mathrm{DCA}\right)=\frac{\mathrm{Total}\;\mathrm{number}\;\mathrm{of}\;\mathrm{aberrant}\;\mathrm{divided}\;\mathrm{cells}}{\mathrm{Total}\;\mathrm{number}\;\mathrm{of}\;\mathrm{divided}\;\mathrm{cells}}\times100$$

*"Total number of aberrant divided cells" refers to the number of cells that exhibit a type of aberration at any dividing stage (prophase, metaphase, anaphase, and telophase).*

$$\%\;\mathrm{of}\;\mathrm{interphase}\;\mathrm{chromosomal}\;\mathrm{aberrations}\;\left(\mathrm{TCA}\right)=\frac{\mathrm{Total}\;\mathrm{number}\;\mathrm{of}\;\mathrm{aberrant}\;\mathrm{cells}\;\mathrm{at}\;\mathrm{interphase}\;}{\mathrm{Total}\;\mathrm{number}\;\mathrm{of}\;\mathrm{cells}}\times100$$

*"Total number of aberrant cells at interphase" refers to the number of cells that exhibit a type of aberration at interphase stage only.*

### Genotoxicity evaluation and DNA degradation assay

The DNA was extracted from liquid N_2_ frozen leaves collected from each treatment using CTAB extraction protocol [30]. The isolated DNA used as a template to amplify inter simple sequence repeats (ISSR) regions within the genome of each sample using universal primers (see Table 1). The PCR reaction was performed using the same protocol of Soliman et al. [31]. Comparison between the ISSR banding profile of each treatment was used as an indication of the percentage of genotoxicity encountered by each treatment. For DNA degradation assay, the gel electrophoresis technique was used. A 7µL of DNA (~ 2µg) extracted from each treatment was incubated with equal amount (7µL) of the corresponding Cr (10 or 50 ppm) whether with or without *Azolla* treatment. While, control extracted DNA was incubated with an equal amount of sterile dist. H_2_O. All samples were incubated at room temperature for 2 h, then the samples were loaded in 1% agarose gel and electrophoresed. The smearing of DNA observed in gel was used as an indication of DNA degradation, modified from Silva et al. [32].

**Table 1:** List of selected ISSR primers, including their codes, sequences, annealing temperature, number of amplified markers, for each primer in each treatment

No.	Primers codes	Sequencing (5′−3′)	Annealing temp. (°C)	No. of markers amplified
1.	HB-14	(GT)6CC	50°C	5
2.	I-844	(CT)8GC		6
3.	I-885	CGTACTCGT(GA)5		6
4.	I-889	AGTCGAGT(AC)5		0
5.	ISSR-5	(ACG)4GAC		3
Total markers amplified				20

### Quantitative gene expression analysis

Leaf tissues were collected from each group and finely ground in liquid nitrogen then the total RNA was extracted using Zymo Direct-Zol RNA Miniprep kit (Zymo Research, R2050) according to manufacturer’s instruction. The isolated RNA was DNase treated to remove any DNA contaminant using DNase I (ThermoScientific cat # EN0521). Successful DNase treatment was confirmed by the absence of any PCR product after 39 cycles using rRNA gene primers and DNase treated RNA as a template. 2 µg of DNase-treated RNA was used for cDNA synthesis with Maxima reverse transcriptase (ThermoScientific) and oligo (dt)_18_ primers (cat# 18064-014, Life technologies-Invitrogen), following the manufacturer’s instructions. The pooled cDNA synthesized from six independent biological replicates were served as a template for the qPCR analysis.

HERA PLUS SYBR green qPCR master mix (cat# 600882, Agilent Technologies-Stratagene) was used to quantify the gene expression. All PCRs were performed in duplicate using a QuantStudio 5 Real-Time PCR System (Applied Biosystems, USA) with cycling parameters: 95°C (2 min) then 39 cycles of 95°C (15 s), and 60°C (20s). The candidate and housekeeping genes were both amplified in the same run. Because rRNA was the housekeeping gene, it was utilized to normalize target gene expression. Melting curve examination of the amplification results verified that the primers produced only one product.

The primer sequences and their IDs that were used are listed in Table 2. The fold changes in relative gene expression were assessed by the comparative Ct method. using 2^−ΔΔCt^ according to Livak et al. [33]

**Table 2:** Primer’s list including their codes, sequences and reference for their sequences used for quantitative gene expression analysis of *V. faba* leaves using real-time PCR.

Description	primer Sequence (5´→3´)	Reference
**S79 (P-type plasma membrane (PM) H+-ATPase)**	F: ACTGGCCATAGCAAAGGAGA	[34]
	R: GAAATACCCCAGCAAATCCA	
**S80 (Calcium-dependent protein kinase sk5-like)**	F: CTCTGCACACACAACCCAAC	
	R: GTCCCATGGATCCTGACAAC	
**rRNA encoding gene **	F: CTTGCAGTCAAGCTCCCTTC	
	R: CCTTGTCCCAAGACAGACCA	

### Statistical analysis

The data represented as a mean of three independent replicas with ± standard deviation (±SD) otherwise stated specifically in the corresponding analysis. To test the significant differences (*p* < 0.05) between the treatment and control means, one-way analysis of variance (ANOVA) companied with Tukey multiple assays was applied in the SPSS program, version 20 for Windows (SPSS Inc, USA). Means with different small letters in tables and figures are indicative of significant differences between samples as determined by the Tukey test at *p* < 0.05. The normality test was performed using Shapiro-Wilk test indicated that the data were normally distributed at (*p* < 0.05).

## Results and discussion

### Efficiency of Cr (VI) phytoremediation from water by* A. pinnata*

The encouraging results obtained from employing *Azolla* as a phytoremediator for Cr in water solution are presented in Table 3. The bioleaching activity of Cr exhibited a dependence on concentration, with a higher removal rate being observed at lower concentrations. At 0.1 ppm, the removal efficacy peaked at 70%, and at 90 ppm, it dropped to 19.53%. The phytoremediation capability of *A. pinnata* against a variety of heavy metals, such as Cr, Cd, Pb, and Ni, at concentrations ranging from 2 to 10 ppm, was confirmed by Rai [35]. The author verified that *A. pinnata* exhibited a high affinity for Cr ion hyperaccumulation, which varied from 70% (treatment with CrO_4_^2−^ at 3.0 ppm) to 88% at 0.5 ppm**.** An additional investigation revealed that the removal efficacy of *A. pinnata* exhibited a decline as the concentration of metals rose from 5 to 25 ppm. Maximum removal efficiency was attained with heavy metal concentrations of 5 ppm and a contact time of 10 days, according to Naghipour et al. [19]. The results of the study suggested that *Azolla* had a significant capability of removing heavy metals from water sources. The rhizofiltration efficacy of *Azolla* in sets of water contaminated with nickel and copper has been previously determined. According to Banerjee & Roychoudhury [36], the authors attributed this phenomenon to the increased discharge of organic acids, such as malic and citric acids, as root exudates. These acids facilitated the scavenging, absorption/adsorption, or sedimentation of heavy metal pollutants in *Azolla*'s roots, while the phyto-stabilization of the toxicant did not impact the overall biomass of the plant. Cr ions can be accumulated by *Azolla* spp., more precisely *A. microphylla*, *A. pinnata*, and *A. filiculoides*, at concentrations ranging from 5000 to 15,000 μg g^−1^. An increase in Cr concentrations led to a more pronounced buildup of the metal in desiccated biomass. Based on the bioconcentration factors (BCF) observed for the three species, which ranged from 243 to 4617, it is plausible that the *Azolla* spp. utilized by Arora et al. [37] could be employed for the remediation of wastewater contaminated with Cr. Phytoremediation comes first among many chemical and physical treatments for wastes or polluted water because it is eco-friendly tool and effectively cheap. This confirmed by Prusty and Satapathy [38], who confirmed that phytoremediation is a visually appealing and tenfold cost-effective alternative technique comparable to physical, chemical, or thermal cleanup procedures. Additionally, it powered by solar energy, and requires little maintenance once established. Phytoremediator plants can be considered biological ores of prized heavy metals, which is an additional beneficial characteristic of phytoremediation. Conversely, this technology is limited by the site's meteorological and geologic conditions, temperature, soil type, and availability to agricultural equipment [39].

**Table 3:** The phytoremediation efficiency of *Azolla pinnata* for Cr from chromium polluted water (0.05 to 90 ppm Cr)

	Cr concentration (ppm) Without ***A. Pinnata*** treatment	Cr concentration (ppm) With ***A. Pinnata*** treatment	phytoremediation efficiency (***E***)(%)
**1.**	0.05	0.05 ± 0.0^f^	0.000
**2.**	0.1	0.03 ± 0.0^f^	70.00
**3.**	0.3	0.19 ± 0.0^f^	36.66
**4.**	0.5	0.29 ± 0.0^f^	42.00
**5.**	10	8.46 ± 0.08^e^	15.40
**6.**	30	23.28 ± 0.06^d^	22.40
**7.**	50	38.03 ± 0.16^c^	23.94
**8.**	70	52.71 ± 0.32^b^	24.70
**9.**	90	72.42 ± 0.80^a^	19.53

The data represent the means of three replicas with ± SD. Means with different small letters indicating statistically significant based on Tukey test at *p* < 0.05

### Effect of *Azolla* treated chromium solutions on* Vicia faba* growth parameters

**Fig. 1:**
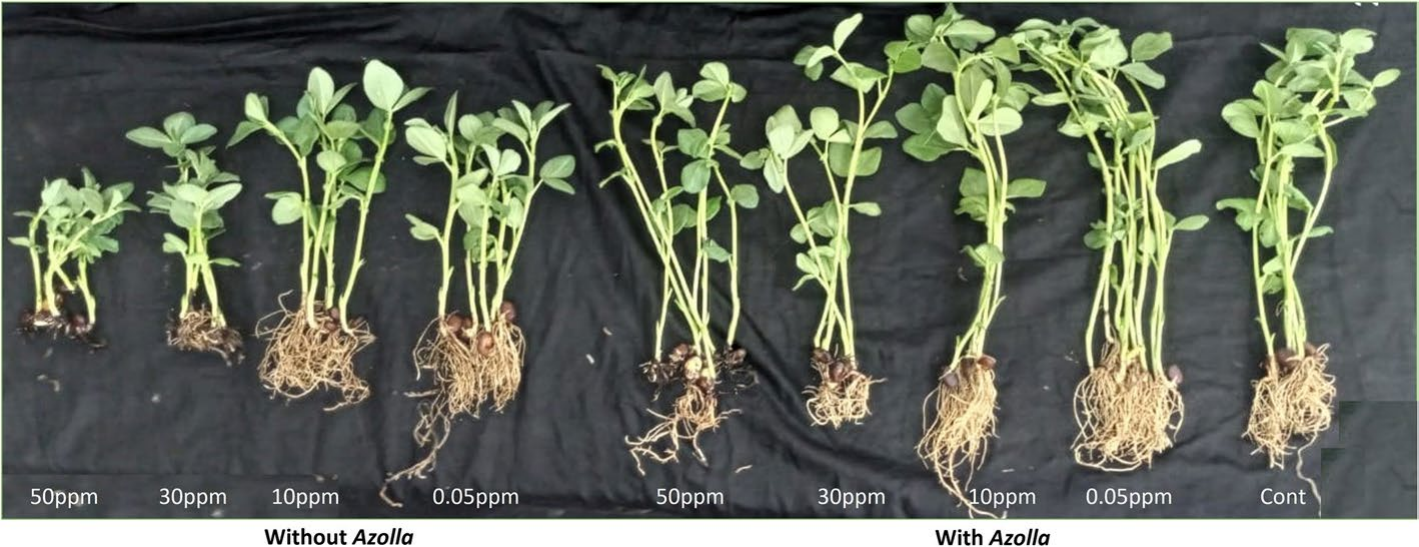
Effect of using *Azolla* (with *Azolla* group) or non-*Azolla* (Without *Azolla* group) treated Cr solutions (0.05, 10, 30 and 50) on the vegetative growth of *Vicia faba* (38 days old) in comparison to control (no-Cr, 0 ppm)

Significant reduction in plants’ growth at different chromium concentrations were observed, more pronounced in the no-*Azolla* groups. Compared with control plants, plants irrigated with chromium solution without prior *Azolla* treatment showed stunted growth with considerable reduction in the measured growth attributes, more significant at 50 ppm (Table 4). The roots were heavily stunted and brownish in color (Fig. 1). The reduction was 69.05% for shoots height, 66.29% shoots fresh weight and 59.55% for shoots dry weight, while was 82.68% for roots length, 71.5% for roots fresh weight and 35.86% for roots dry weight, at 50 ppm of Cr. The *Azolla* treatment significantly mitigates the decline in the measured growth parameters. Significant differences were observed at 50 ppm in compared to the control, whereas no or only minor differences were detected at a concentration of 10 ppm. At 50 ppm Cr that was *Azolla* treated, the reduction in the measured growth parameters was significantly alleviated, reached 23.76 for shoots height, 35.77 shoots fresh weight, and 45.65% for shoots dry weight. Similar alleviation in the growth reduction was observed for the measured roots parameters, reached 67.59 for roots length, and 51.65 for roots fresh weight due to *Azolla* treatment.

**Table 4:** Effect of using *Azolla pinnata* or no *Azolla pinnata* treated Cr solutions (0.05, 10, 30 and 50 ppm) on the growth parameters of *Vicia faba* (38 days old) in comparison to control (no-Cr, 0 ppm), The data represent the means of three replicas with ± SD

Cr conc.	Treatment	Length (Cm)		Fresh weight (g)		Dry weight (g)		No. of leaves
		Shoot	Root	Shoot	Root	Shoot	Root	
**0 (Control)**	***No Azolla treatment***	33.12 ± 1.3^ab^	10.8 ± 1.2^a^	5.34 ± 0.47^ab^	1.51 ± 0.44^a^	0.403 ± 0.10^ab^	0.092 ± 0.016^abc^	5 ± 0.57^a^
**0.05**		19.00 ± 0.0^e^	9.75 ± 0.9^a^	4.40 ± 0.59^bcd^	1.25 ± 0.19^ab^	0.267 ± 0.04^cde^	0.118 ± 0.045^ab^	4 ± 0.00^a^
**10**		21.00 ± 1.8^de^	9.00 ± 0.0^a^	5.05 ± 0.44^ab^	1.50 ± 0.16^a^	0.366 ± 0.03^abc^	0.126 ± 0.026^a^	4 ± 0.570^a^
**30**		15.25 ± 3.3^ef^	3.25 ± 1.2^c^	3.17 ± 0.49^d^	0.79 ± 0.20b^c^	0.278 ± 0.05^cde^	0.090 ± 0.019^abc^	4 ± 0.57^a^
**50**		10.25 ± 0.9f	1.87 ± 0.6^c^	1.80 ± 0.42^e^	0.43 ± 0.26^c^	0.163 ± 0.02^e^	0.059 ± 0.013^c^	4 ± 0.57^a^
**0.05**	***Treatment with Azolla***	37.87 ± 2.4^a^	9.50 ± 0.4^a^	6.00 ± 0.45^a^	1.53 ± 0.15^a^	0.381 ± 0.01^abc^	0.086 ± 0.011^abc^	3 ± 0.57^a^
**10**		34.25 ± 3.0^a^	10.2 ± 0.9^a^	5.85 ± 0.58^a^	1.68 ± 0.29^a^	0.422 ± 0.03^a^	0.125 ± 0.011^a^	4 ± 0.00^a^
**30**		25.37 ± 4.0^bc^	6.75 ± 0.9^b^	4.53 ± 0.92^bc^	0.86 ± 0.17^bc^	0.291 ± 0.02^bcd^	0.073 ± 0.003^bc^	4 ± 0.00^a^
**50**		25.25 ± 3.5^cd^	3.50 ± 1.2^c^	3.43 ± 0.38^cd^	0.73 ± 0.12^bc^	0.219 ± 0.02^de^	0.060 ± 0.002^c^	3 ± 0.57^a^

Means with different small letters indicating statistically significant based on Tukey test at *p* < 0.05

The current results indicated severe effect of Cr on the underground root system, as it is the first site of Cr exposure from soil and the basic location for Cr absorption. Cr(VI) accumulated at the root tip and severely influenced apical dominance by suppressing mitosis and cell elongation in *Arabidopsis thaliana, Brassica campestris, Gossypium hirsutum, Zea mays, and Solanum lycopersicum* [40]. The present finding (see Fig. 1) are consistent with the results reported by Mallick et al. when they exposed *Zea mays* to elevated levels of Cr(VI). Their investigation revealed that this exposure led to a reduction in root length, a brownish color, and few root hairs. The crosstalk between auxin and ethylene may be a factor in the reduced size of roots brought on by Cr [41]. Wakeel et al. [42] demonstrated that the inhibition of primary root growth by Cr is controlled by ethylene modification. Ethylene induced auxin accumulation by regulating AUX1 expression, inhibiting the primary root development. Chromium passes into plants from the underground root and is partially translocated with nutrients to the aerial parts of the plant, where it exerts negative impacts on growth and metabolism. Basit et al. [43] reported that soybeans treated with 0.5 mg L^−1^ chromium and 10 mg L^−1^ in hydroponics and soil cultures, respectively, exhibited a reduction in the dry matter content in the aerial sections. In general, it is assumed that the presence of chromium inhibits plant photosynthesis and consequently disturbs plant growth. High concentration of chromium interrupted many physiological traits in aerial parts, such as transpiration, mineral content, and enzymatic reactions in the plant body, resulting in a significant reduction in leaf area and biomass [13]. As shown in Table 4, the application of *Azolla* for chromium water treatment prior to irrigation resulted in a two-fold increment in both the height and fresh weight of the plants, comparison to those irrigated without *Azolla* treatment*.*

### Effect of *Azolla* treatment on Cr and K accumulation on* Vicia faba*’ root and shoot

**Fig. 2:**
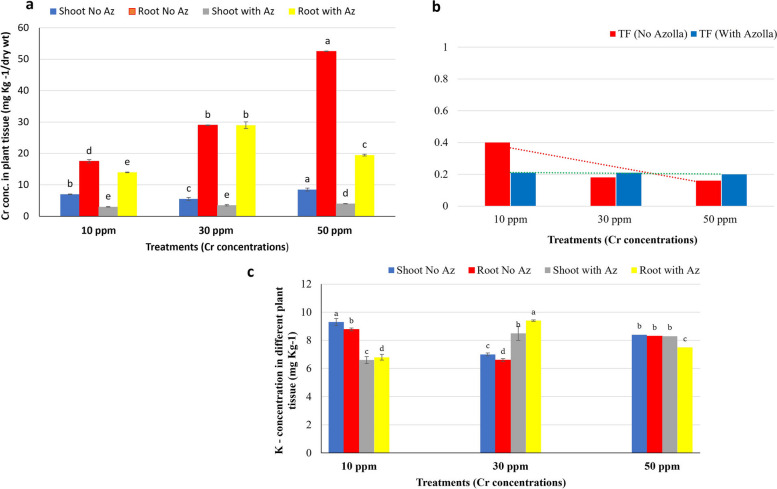
Concentration of Cr and K in root and shoot of *V. faba* irrigated with *Azolla* (with AZ)/or non-*Azolla* (No AZ) treated chromium solution of different concentrations (10, 30 and 50 ppm Cr). **a** The concentration of Cr. **b** translocation factor of Cr. **c** The concentration of K. The data represent the means of three replicas with ± SD. Means with different small letters indicating statistically significant based on Tukey test at *p* < 0.05

Chromium enters plants through the root system and proceeds to the shoot along with other nutrients. Consequently, a greater concentration was observed in the roots; however, the accumulation exhibited a direct correlation with the concentration that was administered. At 50 ppm of Cr, the highest Cr concentrations in the root and shoot were 52.5 and 8.5 mg Kg^−1^, respectively (Fig. 2a). A low translocation factor (TF) (less than 1) for Cr was observed in *V. faba* plants, which was concentration dependent (Fig. 2b). There was an observed inverse correlation between TF and Cr concentration, with values of 0.4 and 0.2 at 10 and 50 ppm Cr, respectively, in the absence of *Azolla* treatment. This result demonstrated the propensity for Cr to accumulate in roots.

Perhaps the reason for Cr's low translocation factor is that it is less mobile in plants compared to other heavy metals. This would account for the greater Cr accumulation in roots as opposed to shoots [44]. Cr translocation and accumulation in plant roots and shoots would be enhanced by increased concentrations of Cr (VI) in irrigation water [4, 45]. As Cr does not play a crucial role in plant metabolic processes, neither a distinct mechanism nor a specific set of carriers are accountable for its uptake [46, 47]. It is highly probable that Cr (III) enters via a passive mechanism, as suggested by Shanker et al. [48]. Alternatively, active absorption may occur via transmembrane protein-mediated anion transport across the plasma membrane, as documented by Appenroth et al. and Cervantes et al. [49, 50].

The present investigation revealed that the application of *Azolla* for water treatment resulted in a reduction of the accumulated Cr in roots and shoots to 19.5 mg Kg^−1^ and 4 mg Kg^−1^, respectively, at 50 ppm Cr. This can be attributed to the ability of *Azolla* to decrease the concentration of Cr in the irrigation water that was utilized. As a result, the overall accumulation of Cr in plant tissue was diminished (refer to Table 3). The administration of *Azolla* resulted in an additional decrease in the TF due to a diminished accumulation dose of Cr in various plant tissues. When rice was intercropped with *Azolla* in flooded soil contaminated with Cd, the amount of soluble Cd in the soil decreased by 37%, resulting in an 80.3% reduction in Cd concentrations in rice grains and a 13.4% increase in yield. This result could potentially guarantee the safety of large-scale double rice cultivation in soil contaminated with Cd [51].

The transportation of water, nutrients, and carbohydrates within plant tissue is facilitated by potassium. It is implicated in the activation of enzymes within the plant, which influences the synthesis of glucose, protein, and adenosine triphosphate (ATP) [52]. Potassium (K) concentrations in both root and shoot of *Vicia faba* exhibited marginal variation across concentrations, but had a substantial relationship with the Cr concentration employed. The root and shoot of the same treatment exhibited comparable concentrations of potassium (K). An increase in K content was observed at 30 ppm Cr (Fig 2c), whereas *Azolla* treatment at 10 ppm Cr decreased the K content in both roots and shoots (6.6 and 6.8 mg Kg^−1^ in comparison to 9.3 and 8.78, respectively, in the absence of *Azolla* treatment). This may be attributed to the fact that *Azolla*, an effective hyperaccumulator of chromium, eliminated approximately 15.4% of the chromium present in a 10 ppm Cr solution (refer to Table 3). The potential consequence of *Azolla* reducing chromium stress on *Vicia faba* plants is a potential decrease in potassium uptake or translocation to the roots and shoots. The potential benefit of *Azolla* at a concentration of 30 ppm Cr to *Vicia faba* plants was the mitigation of chromium stress, which would have enabled the plants to retain excess potassium for critical physiological functions. Conversely, the concentration of K remained constant at 50 ppm Cr regardless of *Azolla* treatment. This may be attributed to the fact that, despite *Azolla*'s marginally higher chromium removal efficiency of 23.94% at this concentration, the *Vicia faba* plants may have been subjected to an excessive amount of chromium stress, which impeded potassium uptake and translocation mechanisms. The potential impact of potassium dichromate (K_2_Cr_2_O_7_) as the chromium source on potassium concentrations in plant tissues should not be ignored. In soil, potassium dichromate dissociates into chromate (CrO_4_^−2^) and potassium (K^+^) ions [53].

### Effect of *Azolla* treated chromium solutions on* Vicia faba*’ physiological attributes

#### Photosynthetic pigments

In accordance with growth traits, increasing chromium concentration from 0.05 to 10 ppm in the irrigated water leads to a non-significant increase in the chlorophyll a (Chl a) compared to the control. However, chlorophyll b (Chl b) content significantly decreased at 0.05 and 10 ppm. At 30 and 50 ppm of chromium, the content of Chl a and b significantly (*p* ˂ 0.05) declined in the leaves of Cr-treated plants compared to control. The highest percentage of decrease was 41.65 and 52.80% for Chl a and Chl b, respectively, at 50 ppm of Cr. The total chlorophyll content showed the same decline at 10, 30 and 50 ppm of Cr (Table 5).

**Table 5:** Effect of using *Azolla pinnata* or no-*Azolla pinnata* treated Cr solutions (0.05, 10, 30 and 50 ppm) on the Chl a and b content (mg g^−1^ Dwt), total Chl content and Chl a/Chl b ratio of *Vicia faba* leaves (38-d-old) in comparison to control (no-Cr, 0 ppm), The data represent the means of three replicas with ± SD

Cr	Treatment	Chl a	Chl b	Total Chl	Chl a/Chl b
**0 (Control)**	***No Azolla treatment***	8.092 ± 0.07^**a**^	4.524 ± 0.35^**a**^	12.61	1.788
**0.05**		8.435 ± 0.44^**a**^	4.250 ± 0.35^**abc**^	12.96	1.984
**10**		8.680 ± 0.28^**a**^	3.120 ± 0.07^**d**^	11.80	2.782
**30**		6..419 ± 0.27^**b**^	3.185 ± 0.16^**d**^	9.604	2.015
**50**		4.721 ± 0.24^**c**^	2.135 ± 0.08^**e**^	6.856	2.211
**0.05**	***Treatment with Azolla***	8.718 ± 0.26^**a**^	4.500 ± 0.28^**a**^	13.21	1.937
**10**		8.815 ± 0.24^**a**^	4.420 ± 0.20^**ab**^	13.23	1.994
**30**		6.412 ± 0.37^**b**^	3.580 ± 0.33^**cd**^	9.992	1.791
**50**		6.540 ± 0.17^**b**^	3.615 ± 0.29^**bcd**^	10.19	1.809

Means with different small letters indicating statistically significant based on Tukey test at *p* < 0.05

Previous studies have demonstrated that an increase in Cr concentrations led to a reduction in the amount of photosynthetic pigments present, while low Cr concentrations resulted in a positive increase in pigment content [54]. The decrease in chlorophyll levels observed in plants irrigated with Cr could potentially be attributed to the inhibition of specific enzymes implicated in the biosynthetic pathway, which disrupts chlorophyll production and biosynthesis [44]. At 400 ppm, Ni and Cr showed a strong detrimental influence, inhibiting ALA-D activity by up to 50% and dramatically lowering total chlorophyll content in maize [55]. Increasing Cr content up to 20 mg Kg^−1^ resulted in a significant decrease in chlorophyll (a, b, and total chlorophyll) due to impaired mineral nutrient absorption and translocation [56, 57]. This nutritional shortage may be result from metal translocation to shoots at high concentrations. Plant photosynthetic capacity is directly proportional with the total photosynthetic pigment (total Chl) viability, so reduction in chlorophyll at high Cr concentrations impaired photosynthesis process, resulting in slower growth rates.

By using *Azolla* as a phyto-tool to remediate Cr from contaminated irrigation water, significant (*p ˂ 0.05*) increase in Chl a and b was observed at 50 ppm when compared to no-*Azolla-*treated Cr solution at the same concentration. Moreover, *Azolla* treatment recovered the ratio between Chl a and b to become near the control value of 1.788. *Azolla* as a bioremediation tool can efficiently remove Cr from contaminated irrigation water, offering an efficient, low cost and environmentally-sustainable technology for Cr-polluted water [58]. While *Azolla* has no direct mechanism for chlorophyll restoration under Cr stress, its ability to remove contaminants from environments supports chlorophyll content in plants under Cr stress. Chlorophyll is considered a determinant factor for photosynthetic capacity and consequently plant development [59], reflecting the recovery of growth metrics.

#### Antioxidant defense response activity

Plants exposed to heavy metal stress experienced many defence mechanisms, including both enzymatic and non-enzymatic. Peroxidase and catalase are basic enzymes involved in reactive species detoxification under Cr contamination. Peroxidase enzyme activity significantly increased with the elevation of Cr concentration in growth medium from 10 to 50 ppm, comparable with control, reaching its highest activity 67.51 µg g^−1^ Fwt sec^−1 ^at 50 ppm. However, catalase activity showed no significant response towards the increased Cr concentration. Total antioxidant capacity (TAC) revealed a significant elevation only at the highest concentration of Cr compared to the control (Table 6).

**Table 6:** Effect of using *Azolla pinnata* or no-*Azolla pinnata* treated Cr solutions (0.05, 10, 30 and 50 ppm) on the peroxidase (POX) and catalase (CAT) activity (µg g^−1^ fwt sec^−1^) and total antioxidant capacity (mg Asc g^−1^ Fwt) of *Vicia faba* leaves in comparison to control (no-Cr, 0 ppm), The data represent the means of three replicas with ± SD

Cr	Treatment	POX	CAT	Antioxidant capacity
0 (Control)	No Azolla treatment	7.93 ± 0.57^f^	12.17 ± 1.64^abc^	2.603 ± .0.38^bc^
0.05		9.66 ± 0.26^f^	8.70 ± 0.75^cd^	2.99 ± 0.02^ab^
10		30.33 ± 0.70^d^	14.07 ± 0.70^ab^	2.75 ± 0.20^abc^
30		49.68 ± 0.07^b^	13.72 ± 1.10^ab^	2.52 ± 0.05^bc^
50		67.51 ± 3.29^a^	15.03 ± 2.5^a^	3.56 ± 0.01^a^
0.05	Treatment with Azolla	13.95 ± 0.05^e^	8.88 ± 0.24^cd^	2.42 ± 0.49^bc^
10		30.40 ± 0.0.40^d^	7.96 ± 1.55^d^	1.97 ± 0.10^c^
30		37.39 ± 0.50^c^	10.69 ± 0.98^bcd^	2.21 ± 0.51^bc^
50		39.78 ± 0.25^c^	11.59 ± 0.25^abcd^	2.73 ± 0.2^bc^

Means with different small letters indicating statistically significant based on Tukey test at *p* < 0.05

The outcomes of our study were consistent with those of Sharma et al. [60], who observed an increase in the activity of various peroxidases, such as guaiacol peroxidase (POD), ascorbate peroxidase (APOX), and glutathione peroxidase (GPOX) by increasing Cr in the agricultural medium. Conversely, a reduction in the catalase (CAT) activity was noted in the seedlings subjected to Cr stress. When Cr concentrations in soil are high, the activity of NADPH oxidases consumes cytosolic NADPH to form free radicles O_2_. These radicles are then converted to H_2_O_2_ via the action of the superoxide dismutase enzyme (SOD) [61]. These free radicals are extremely reactive and destructive to cellular components. As a result, plants have evolved specialized defence mechanisms, including the biosynthesis of antioxidant enzymes [3, 4].

By using chromium solutions pre-treated with *Azolla*, the peroxidases activity of *V. faba* leaves decreased by 24.7 and 41% at 30 and 50 ppm of Cr concentrations, respectively. Conversely, TAC was significantly reduced only at *Azolla* treated 50 ppm Cr solution compared to untreated one. On the other hand, the *Azolla* treatment had no effect on CAT enzyme activity under different concentrations of Cr. The rate of detrimental oxidative stress induction is reduced in chromium irrigation water treated with *Azolla i*n comparison to untreated chromium irrigation water (Table 6). While there is no literature investigating an indirect role of *Azolla* in reducing the harmful effects of Cr by removing it from the cultivation medium and restoring the redox balance of the cell. The current study shows how the peroxidases activity, as a part of the antioxidant defence system, was reduced by *Azolla* and promoting plant growth and biomass. Similarly, Dubey et al. [62] found that the augmented activities of SOD, GST, GPX, and APX enzymes in rice were enhanced to support the scavenging of elevated free radicals by Cr(VI) application.

#### Effect of *Azolla* treated chromium solutions on *Vicia faba’* Cytogenetics

**Fig. 3:**
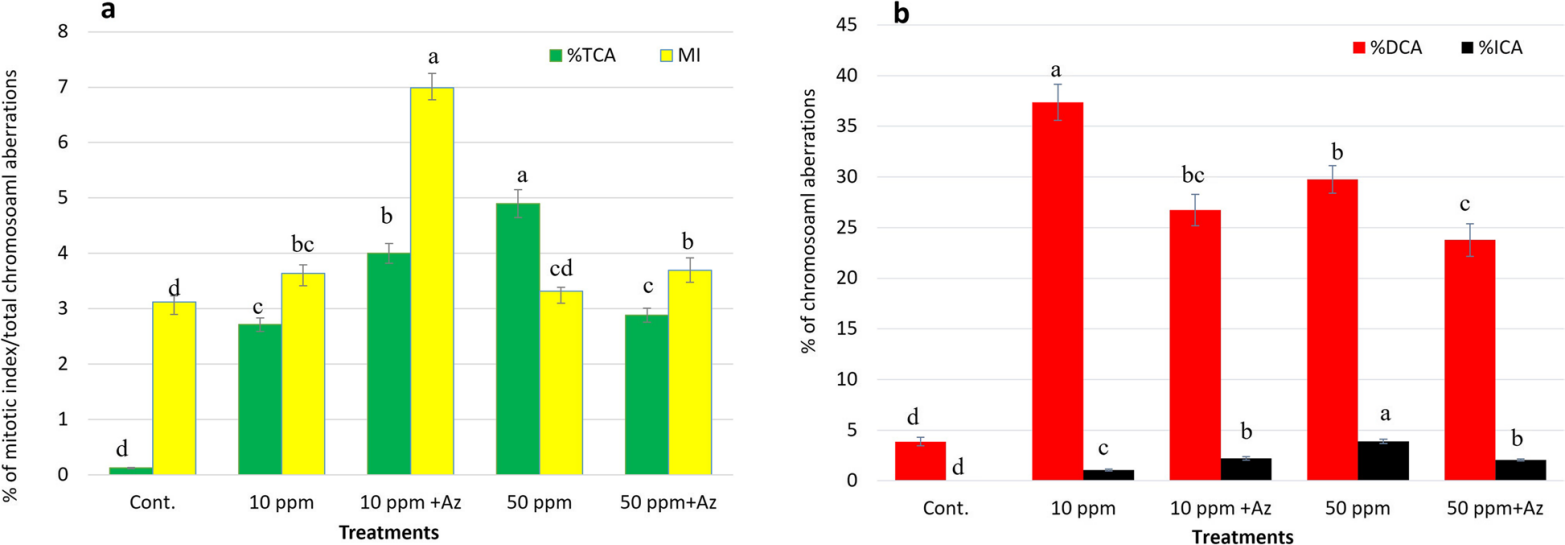
Effect of different concentrations of Cr (10 and 50 ppm) with and without *Azolla* treatment on *Vicia faba* root apex **a**: Mitotic index (MI) and percent of total chromosomal aberrations (%TCA). **b**: percentage of aberrant cells; the (%DCA) refers to chromosomal aberrations in the dividing cells while (ICA) refers to chromosomal aberrations in the non-dividing cells (interphase). The data represent the means of ~ 3000 -5000 cells with ± SD. Means with different small letters indicating statistically significant based on Tukey test at *p* < 0.05

**Fig. 4:**
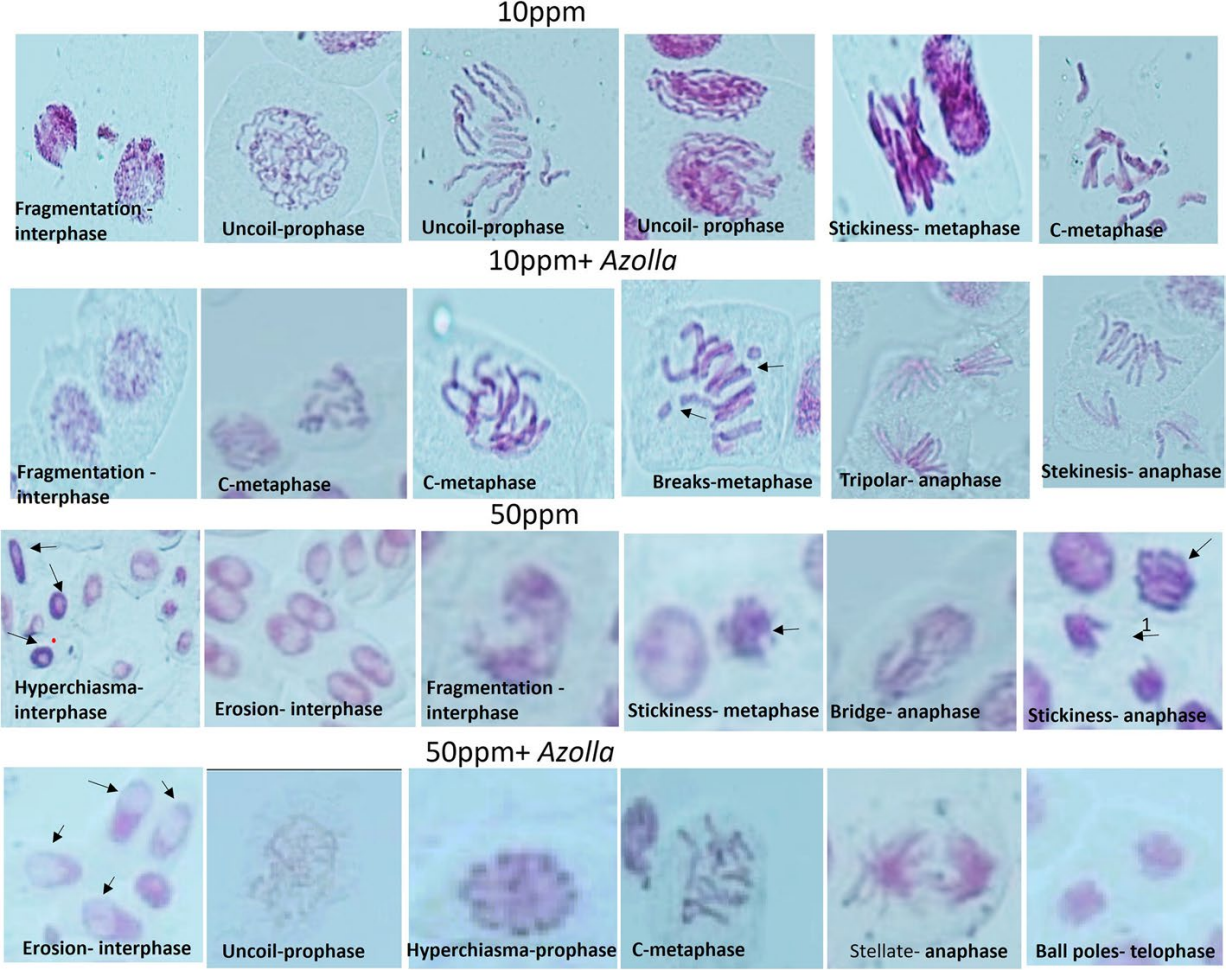
Examples of chromosomal aberrations induced in *Vicia faba* root tips irrigated with 10 or 50 ppm chromium solution without *Azolla* or with *Azolla*

Altered mitotic index (MI) was observed in the plants watered with chromium solution at different concentration. Without *Azolla* treatment, at 10 ppm, the MI was (3.6±0.15) combined with an increase in the total chromosomal aberration (TCA) (2.15±0.12) in comparison to MI of 3.1±0.11 and TCA of 0.126±0.013 for controls. At 50 ppm, a comparable MI was noticed with an even higher TCA of 4.9±0.25. Although, a higher MI was observed in case of Cr treatment, but this was concomitant with an increase in the TCA, which could explain the boost in the MI to compensate the mutated cell in favor of growth. In case of *Azolla* treatment, the highest percentage of MI 6.99±0.25 was observed, with a 32.5% increment in the TCA compared to those observed without *Azolla* treatment at the same concentration of Cr. At 50 ppm, the presence of *Azolla* reduces the effect of this high concentration of Cr, allowing the cell to respond as if challenged with 10 ppm of Cr (Fig. 3a). Phyto-genotoxic effect of Cr at various concentrations (1, 3, 6 and 12 ppm) was evident through inhibited root length, protein concentration, MI and increased TCAs of *Allium cepa* [63]. The highest percentage of TCA observed was a result of chromosomal aberration at the dividing stages (DCA). *Azolla* treatment had reduced the DCA% by 39.85 and 25.1% at 10 and 50 ppm, respectively, in comparison to the same concentrations without *Azolla*. Stekinesis, C-metaphase, disturbed anaphase, breaks and uncoiled prophase were the most common aberrations detected. Generally, at interphase, a low percentage of chromosomal aberrations was observed, reaching a maximum at 50 ppm, which was reduced by 69.79% with *Azolla* treatment at the same concentration (Fig. 3b). Fragmentation, hyper-chiasma, and erosion were the most common aberrations detected at interphase (Fig. 4).

The mitotic index shows how often cells divide, which is crucial in determining how fast roots grow and how well anti-mitotic agents work [64]. The MI of onion and black seed plants reached a maximum 6h after treatment with a concentration of 50 ppm Cr, whereas it was at its lowest at 24h after treatment with a concentration of 2000 ppm of Cr. This indicates that Cr(VI) had a dose-and time-dependent effect on root growth rate and the MI [65]. Similar to the current findings, stimulatory effect on the root growth and cell division of *Amaranthus viridis* L. was noticed upon exposure to 10^−5^ M Cr(VI). However, a reduction was concomitant at a higher concentration of 10^−3^ M Cr(VI) [66]. The proportion of aberrant cells increased with the duration of exposure or Cr concentration increased. The possible pathways of Cr-induced genotoxicity may involve the interaction of the metal with DNA, causing DNA damage, cell cycle arrest, and polyploidization [67, 68]. One way *Azolla* may enhance plant growth is by increasing the mitotic index, which is a measure of cell division rate. Disruption of the endoplasmic reticulum by Cr(VI) may have caused disassembly of microtubule (MT), and MT damage is thought to underlie the occurrence of altered cytokinesis cycles, mitotic abnormalities, and chromosomal aberrations. The mitotic aberrations in *Lens culinaris* root cells were caused by (MT) overstabilization rather than MT depolymerization. MT bundle arrangement was affected in both mitotic and interphase root cells, and Cr(VI)-induced tubulin accumulation was occasionally located in the nucleoplasm of root cell nuclei. Acetylated α-tubulin (an MT stability marker) is enhanced after Cr(VI) treatment as observed by western blotting, and Cr(VI) can overcome the depolymerization ability of oryzalin (an MT depolymerization agent), further demonstrating the stabilizing effect of MT [69].

Plants mitigate the deleterious effects of Cr through two important strategies: avoidance and tolerance. Metals avoid their entry into the plant by binding to mycorrhizae for immobilization or complexation with organic matter secreted by the roots, while metals entering the plant would activate tolerance mechanisms [70]. Cr(VI) is converted to Cr(III) in the AMF structure [71, 72]. Metal stores were isolated in subcellular compartments (vesicles) of fungal cells, limiting the translocation of Cr from roots to shoots and limiting their access to sensitive sites such as mitochondria and chloroplasts [73], and organic ligands complex with metals for molecular regulation. Otherwise, *Azolla* roots can reduce Cr(VI) to Cr(III) through chemical or reductase-mediated reduction, reducing the toxicity of Cr(VI) [74].

#### Effect of *Azolla* treated chromium solutions on *Vicia faba’* Genomic integrity

**Fig. 5:**
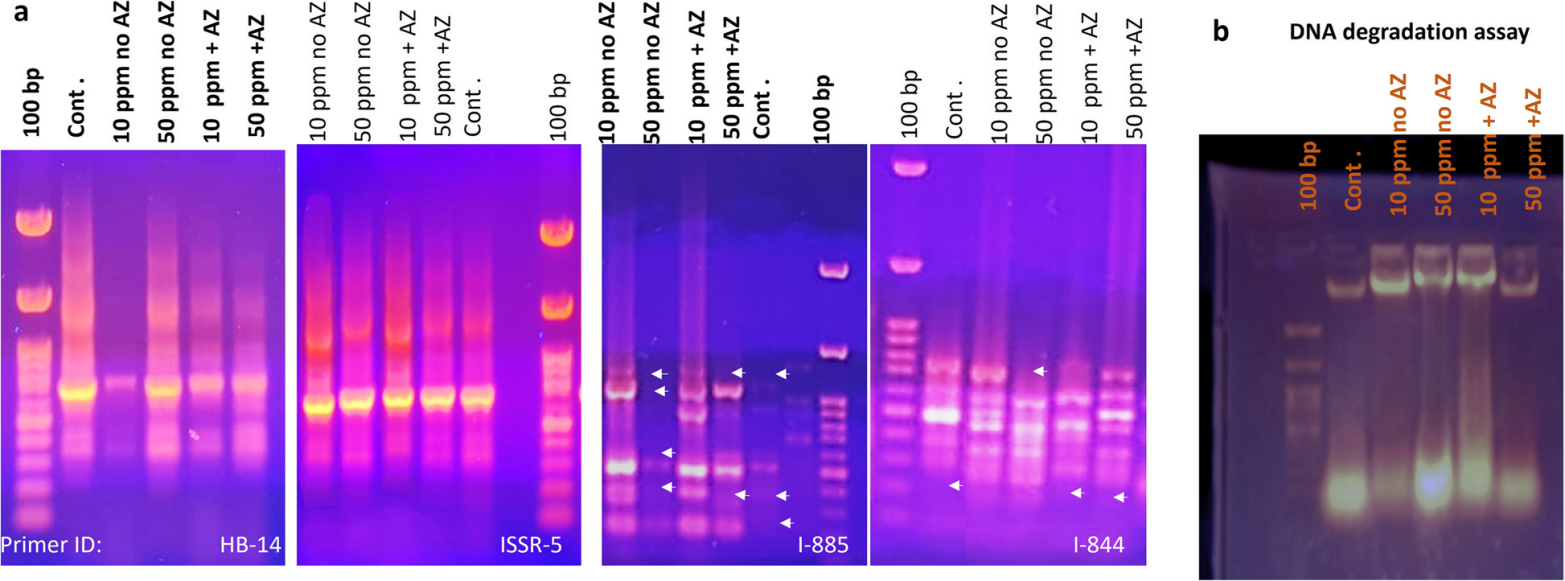
**a** Genetic assessment using ISSR profiling on *Vicia faba* plant irrigated with different concentrations of Cr (10 and 50 ppm) with and without *Azolla* treatment in comparison to control plants (irrigated with tap water without Cr). arrows refer to changes in PCR profile. **b** DNA degradation assay for the same plants. Labels are written on the images

To evaluate the impact of Cr treatment with or without *Azolla* on *Vicia faba* plant’s DNA integrity, two assays were performed: the ISSR and DNA degradation assays. The ISSR is a PCR-based assay that uses universal primers to span the whole genome, targeting the inter-sequence simple repeats using the abundant microsatellite regions as binding sites for the primers. This technique is well adopted to study the genetic identity, genetic diversity, genotoxicity and genetic stability [31, 75–77]. It was used as an indication of genotoxicity induced by various nanoparticles on the callus of *Salvadora persica* [76]. On the other hand, ISSR fingerprinting confirmed the genetic stability of encapsulated seeds of *Cannabis sativa* L. during in vitro multiplication and storage for 6 months under different growth conditions [75]. Each primer produced a unique set of amplification products ranging in size from about 180 bp to 1400 bp (Table 1). The current findings suggest that the ISSR profile underwent minimal changes as a consequence of various treatments. The majority of the detected changes were observed at 50 ppm Cr, where primer I-844 scored a missed allele, in the presence of *Azolla*, the same profile as the control was maintained. At the same concentration, primer I-885 scored two alleles, while four amplifiable alleles were identified when *Azolla* was present. These findings indicated that *Azolla* had the potential to repair DNA (Fig. 5a). Moreover, the DNA degradation assay indicated DNA degradation, as observed by DNA tailing, for the 50 ppm Cr-treated plants upon electrophoresis. In comparison, the *Azolla* treated 50 ppm Cr abolished this degradation, and a visible intact DNA band was observed that was comparable to the control DNA (Fig. 5b). This result suggested a negative consequence of 50 ppm Cr on plant DNA integrity, while *Azolla* treatment ameliorates this negative impact. This degradation may result from the generation of prooxidants generating ROS that target DNA, causing its damage. The *Azolla* treatment effectively reduces DNA degradation, possibly by reducing Cr concentration by its adsorption [32].

#### Effect of *Azolla* treated chromium solutions on *Vicia faba’* gene expression

**Fig. 6:**
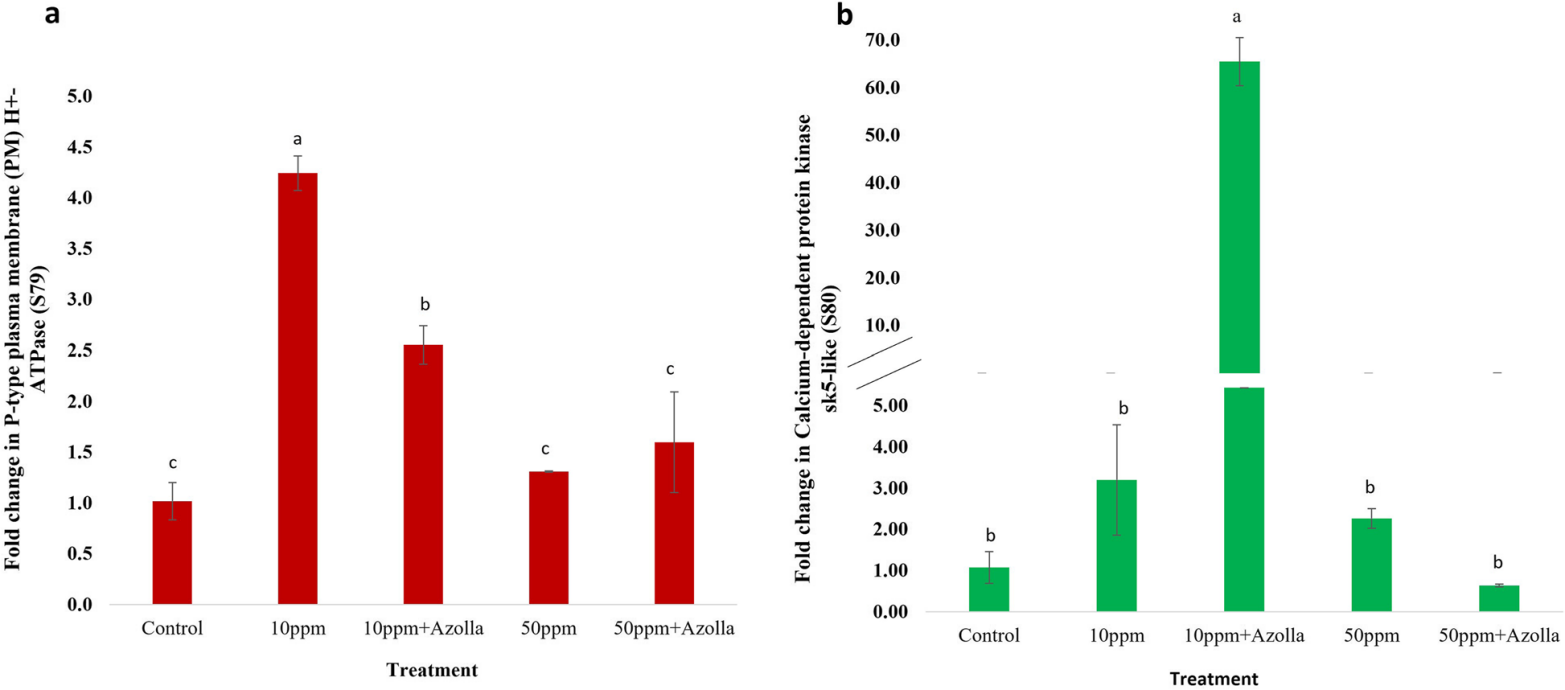
The fold changes in gene expression level of *Vicia faba* shoots irrigated with 10 or 50 ppm Cr (VI) with or without *Azolla* treatment in comparison to control (0 ppm- Cr). **a** S79 encodes PM H+-ATPase and (**b**) S80 encodes Calcium-dependent protein kinase CDPK5-like. The data represent the means of three replicas with ± SD. Means with different small letters indicating statistically significant based on Tukey test at *p* < 0.05

An altered gene expression profile was observed for S79 gene, which belongs to Plasma Membrane transport proteins encoding (PM-type H^**+**^-ATPases), upon Cr treatment (Fig. 6a). Members of this family use the energy from ATP hydrolysis to pump protons (H^+^) across cell membranes. This creates electrochemical gradients that drive secondary transport processes [80]. In plants, PM-type H^**+**^-ATPases play crucial roles in mineral uptake, pH homeostasis, stomatal movement, and adaptation to environmental stresses [78].

A 10 ppm Cr treatment significantly induces the level of expression of the PM-type H^**+**^-ATPase gene four-fold over the control, while 50 ppm non-significantly altered the expression, suggesting dose dependent expression. Chromium (Cr) induces oxidative stress by increasing reactive oxygen species (ROS) production, inhibiting photosynthesis and causing growth inhibition [79]. To cope with Cr stress, plants activate detoxification mechanisms by maintaining the active transport of ions across the PM through PM H^**+**^-ATPase. This helps to refill the loss of essential substances in repair processes and to remove excess toxic Cr ions from the cytoplasm to the outside of cells [80]. Therefore, the transcript level of genes involved in mineral transportations, such as *OsNRAMP1*, *OsRT1*, *OsHMA3*, and *OsLCT1*, was upregulated in Cr-treated rice cultivar Giza 181 plants. A higher level was observed upon elevated CO_2_ (eCO_2)_ exposure compared with untreated plants, while a reverse trend was observed in the cultivar Sakha 106 plants treated with eCO_2_. This suggests cultivar-specific responses, which were inversely proportional to the level of Cr accumulation in both cultivars, especially at 400 mg Cr kg^−1^, suggesting a mitigation effect of eCO_2_ on Cr phytotoxic effect [79]. Long-term Cd stress for 6 days stimulates the expression of genes encoding PM H^+^-ATPases in cucumber roots, enhancing repair processes and inducing Cd stress tolerance [80, 81]. Upregulation of some isoforms of PM H^+^ATPase, such as CsHA2, CsHA4, and CsHA8, in cucumber roots was observed to be involved in brassinosteroids-induced Cd stress tolerance [82]. Similarly, Cd stress for five or ten days in rice increased proton pump activity [83].

*Azolla* treatment significantly enhances the expression level to two-fold at 10 ppm treatment, albeit to a lesser extent compared to those without *Azolla.* This effect may be attributed to *Azolla* reducing Cr concentration available to the plant, thus reducing detoxification mechanisms generated by PM H^+^-ATPase pump due to less amount of Cr. A similar reduced effect was observed upon AG aminoguanidine treatment for cucumber seedlings under Cd stress. AG is a diamine oxidase "DAO" inhibitor involved in the activation of PM H^+^-ATPase upon Cd stress [84].

On the other hand, maintaining the transcript level of the PM H^+^-ATPase gene in response to 50 ppm Cr with or without *Azolla* could be an adaptive mechanism to conserve energy, since the gradient cannot be maintained due to Cr inhibition. This also, confirms the effectiveness of *Azolla* to phytoremediate Cr at low concentrations, i.e.10 ppm. In cucumber seedling roots, a two-hour treatment with Cd or Cu (10 and 100 μM) inhibited PM H^+^-ATPase activity [80]. Different concentrations of different heavy metals showed altered PM H^+^-ATPase activity, in which 10 to 100 µM of Hg, Cu, Cd, Zn, and Pb inhibited the activity, while activation was observed at 1 mM of Cd and Zn, with no activation for Hg, Cu, and Pb [85]. A decrease in ATPase activity by in vivo Cd treatment reached 30% and 90% in wheat and sunflower, respectively [86], suggesting metal-dependent specific response for the PM H^+^-ATPase gene.

Similar to the current findings, it was found that treating *Arabidopsis thaliana* with different concentrations of copper (5 to 20 µm) for 72 hours inhibited AHA2 expression; the predominant proton pump gene in the roots, but had little effect on AHA1 and AHA5. In contrast, treatment of the same plants with Cu for a longer period of 15 days did not alter AHA2 gene expression in comparison to controls, suggesting time dependent response [87].

The CDPK5 gene expression was marginally upregulated at 50 ppm (Fig. 6b), compared to 10 ppm Cr. The generation of ROS and the accumulation of Ca^2+^ by Cr(VI) stimulate the activity of Ca^2+^ signaling proteins, including CDPK [88]. Different concentrations of heavy metals, including Cr(VI), Cu, and Cd, induced the expression of genes encoding protein kinases (DUF26, RLCK, and LRK10L-2) and transcription factors (WRKY and Apetala2), according to microarray data analysis and qPCR validation. In-gel kinase assay showed increased 47- and 49-kDa CDPK activities at 100–400μM Cr(VI), exceptionally, less activation for the 49-kDa CDPK was observed at 400 μM. At 3 hours, transcripts of OsRLCK-VIIa, OsDUF26-If, and OsLRK10L-2 were significantly elevated; at 12 hours, these transcripts decreased in a dose-dependent manner. OsDUF26-If was consistently upregulated in a dose-dependent manner up to 200 M, and to a lesser extent at 300 M, similar to our findings [89]. By phosphorylating NADPH oxidases, CDPKs, such as StCDPK4 and StCDPK5, may regulate Cr(VI)-induced ROS production in the plasma membrane [90]. Furthermore, intracellular Ca^2+^ and adaptive mechanisms are induced by Cd via CDPKs [91].

*Azolla* treatment resulted in the most significant induction at 10 ppm Cr; conversely, a reduction was observed at 50 ppm Cr, which may have been caused by *Azolla* adsorption of a lower concentration of Cr. The growth inhibition of rice caused by Cr(VI) stress was similarly alleviated by exogenous proline application. The proline caused a decrease in the cytoplasmic Ca^2+^ and Cr concentrations as well as activation of the Ca^2+^-dependent signaling pathway that follows. Up to a supplemented concentration of 8 mg L^−1^ Cr, the expression of OsCDPK4, 18, 7, 11, and 15 was significantly upregulated in the shoots of rice plants treated with Cr and proline compared to the corresponding Cr-treated plants. Conversely, at a concentration of 16 mg L^−1^ Cr, the expression of OsCDPK12 and 23 was observed to be significantly downregulated in the shoots of Cr and proline-treated rice plants. Proline enhances Cr tolerance in rice via interaction with Ca^2+^-dependent signaling pathways, suggesting similar mode of action in *Azolla* treated plants [92].

## Conclusion

The primary innovation of the current study is that in addition to evaluating the remediation capacity of *A. pinnata* for Cr, the current study investigated the downstream effects on a test plant (*Vicia faba* L) at various levels of biological organization, integrating molecular, cytogenetic, biochemical, and physiological analyses. Using of *Azolla* treated chromium polluted water in irrigation significantly mitigated the substantial retardation of vegetative growth attributes caused by chromium stress through modification of physiological mechanisms. One of these metabolic changes was that Cr adversely affects the photosynthetic pigments, mainly at 50 ppm. *V. faba* tends to accumulate more Cr in roots (TF˂1), causing a significant retardation of its growth and development. Translocated Cr from roots to shoots enhances oxidative stress and consequently, antioxidant enzymes activity represented in POX and antioxidant capacity that peaks at 50 ppm in a defensive manner. In comparison to non-*Azolla* Cr-stressed plants, *Azolla* treatments for chromium-polluted water resulted in enhanced mitotic indices and reduced total chromosomal aberrations in the dividing cells. This may account for the observed increase in growth. Nonetheless, 50 ppm Cr induced DNA degradation that was reversed following *Azolla* treatment, indicating that *Azolla* treatment may possess DNA repair properties. This may be related indirectly to the decreased Cr concentration caused by *Azolla* treatment. The levels of expression of calcium-dependent protein kinase CDPK5-like gene and PM-type H^+^-ATPase were generally increased in response to Cr stress. However, the extent of expression variation was specific to each treatment type and dose, suggesting that Cr significantly disrupted the typical functions of *Vicia faba* genes' and induced the upregulation of genes associated with stress regulation and adaptation to Cr stress.

The current findings highlight the possible use of a new eco-friendly approach, employing *A. pinnata* in phytoremediation of Cr from aquatic environments, which may replace the chemical remediation strategy, minimizing the negative concerns associated with it. Additional research is necessary to clarify the practical field applicability and long-term efficacy of *A. pinnata* in chromium phytoremediation. Furthermore, it is imperative to establish and verify secure and effective protocols for the management and processing of Cr-laden *A. pinnata* biomass following remediation in order to reduce potential environmental hazards. Moreover, the potential synergistic interactions between *A. pinnata* and indigenous soil microbiota in chromium remediation present an intriguing opportunity for future research to further our comprehension of plant-microbe interactions in the context of heavy metal stress and remediation.

## Data Availability

The relevant datasets supporting the results of this article are included within the article.

## Abbreviations

CA: Chromosomal aberrations
CAT: Catalase
Cr: Chromium
CTAB: Cetyltrimethylammonium bromide
CDPK: Calcium‐dependent protein kinases
ISSR: Inter simple sequence repeats
MI: Mitotic index
PM-type H+-ATPases: Plasma Membrane transport proteins encoding PM-type H^**+**^-ATPases
POX: Peroxidase
TAC: Total antioxidant capacity
ROS: Reactive oxygen species

## Declarations

### Experimental research and field studies on plants

“All relevant institutional, national and international guidelines and legislation were compiled or adhered to the production of this study”.
